# Positive drug test trends in fatally-injured drivers in the United States from 2007 to 2017

**DOI:** 10.1186/s13011-019-0228-z

**Published:** 2019-10-25

**Authors:** Sunday Azagba, Keely Latham, Lingpeng Shan, Fares Qeadan

**Affiliations:** Department of Family and Preventive Medicine, Division of Public Health, University of Utah School of Medicine, 375 Chipeta Way, Suite A, Salt Lake City, UT 84108 USA

**Keywords:** Drugged driving, Substance use, Administrative dataset, Positive drug test

## Abstract

**Background:**

The last two decades have seen tremendous changes in the U.S. environment surrounding drugs. Driving under the influence of drugs is a growing public health hazard. The present study examined trends in drug involvement in fatally-injured drivers in the U.S.

**Methods:**

Data were drawn from the 2007–2017 Fatality Analysis Reporting System. Cochran–Armitage tests were performed to assess the statistical significance of changes in the yearly prevalence of positive drug tests in fatally-injured drivers over time. In addition, analyses were stratified by sex, race, and age.

**Results:**

The yearly prevalence of positive drug tests in fatally-injured drivers increased significantly from 20.7% in 2007 to 30.7% in 2017, with results showing a higher prevalence among males, those aged 21–44, and Whites. The gap between Blacks and Whites narrowed in 2017. There was a decline in the yearly prevalence in all age groups between 2016 and 2017, although the decrease in the 21–44 age group was much smaller than other age groups. Among drivers who tested positive for drugs, 34.6% had a blood alcohol concentration (BAC) above the threshold of per se evidence for impaired driving, and 63% had a BAC below the threshold.

**Conclusions:**

Our results indicate that the overall yearly prevalence of fatally-injured drivers who tested positive for drugs increased significantly from 2007 to 2017, with similar results found for subgroups. Findings further highlight that drugged driving remains a public health priority, and more action is needed to stem this disturbing trend.

## Background

The use of certain drugs is on the rise in the United States. A 2016 study found that marijuana use has increased steeply in the U.S. since 2005 [1]. Additionally, opioid prescriptions have increased significantly in the last three decades, with hydrocodone more than doubling and oxycodone increasing over five-fold from 1999 to 2011 [2]. Heroin, another highly addictive opioid, has seen increases across both men and women, most age groups, and all income levels [3]. Beyond the adverse health outcomes related to drug use, one negative behavior consequence is a heightened risk of traffic crashes [4]. An estimated 12.8 million people aged 16 or older in the U.S. drove under the influence of substances (i.e. marijuana, cocaine (including crack), heroin, hallucinogens, inhalants, or methamphetamine) in 2017 [5], and motor vehicle crashes are a major contributing factor to unintentional injury mortality in the United States [6].

Evidence from the extant literature on the effect drugs have on driving ability has produced mixed results [7, 8]. Findings from several laboratory studies suggest that cannabis can negatively affect neurocognitive functions that may impair driving. These studies have found that reaction time [9, 10], time/distance perception [11], and equilibrium [12] are all impacted by cannabis use. Previous studies suggest that recent cannabis smoking is associated with considerable driving impairment, especially in occasional smokers [13], and that cannabis use is associated with an increased risk of vehicle crashes, particularly fatal crashes [14]. Similarly, studies have associated opioid use with impaired driving. Opioids have been found to impair driving-related psychomotor function, and the risk of injury or death in an accident is increased while using opioids [15, 16]. A recent report found that the use of prescription opioids was associated with the initiation of vehicle crashes [17]. Other opioids such as heroin can cause drowsiness, weakened mental function, difficulty concentrating, and reduced coordination [18, 19], which impact driving ability.

In contrast, some studies were inconclusive or failed to find a link between drug use and driving ability [20–22]. Prior findings have indicated that low concentrations of marijuana compounds in blood serum may decrease the rate of crashes, perhaps due to effective compensation behavioral strategies that have been reported in marijuana smokers [20]. Previous results have suggested that regular use of opioid medications did not significantly impair coordination, perception, cognition and behavioral outcomes related to driving, although the authors recognized the need for research beyond the pilot study conducted [21]. Similarly, a recent systematic review found no significant effect of regular therapeutic opioids on psychomotor skills related to driving ability [22].

Overall, the research on drugged driving remains limited relative to the vast body of research that has extensively documented the involvement of alcohol in fatally-injured drivers. Too, the environment surrounding drugs has changed in recent years in the U.S., including the passage of increasingly liberal marijuana usage laws [23] and the ongoing opioid epidemic [24]. As a result, there are concerns about further escalation of drug use and impaired driving [25, 26]. Recognizing this possible link, some states have enacted per se drugged driving laws, which set maximum concentrations for various substances at or above which it is unlawful to drive or operate various forms of vehicles. However, further research on the relationship between drugged driving and fatally-injured drivers is needed.

The objectives of the current study are (i) to examine trends in drug involvement in fatally-injured drivers in the U.S., (ii) examine trends between drug involvement and fatally-injured drivers by key demographic characteristics (sex, race and age) associated with higher crash risk, and (iii) examine whether fatally-injured drivers testing positive for drugs also tested positive for alcohol. Findings from this study are particularly timely and relevant, especially given the rapidly shifting environment and norms surrounding substance use.

## Method

Data used in this study came from the 2007–2017 Fatality Analysis Reporting System (FARS), which is compiled by the National Highway Traffic Safety Administration (NHTSA). FARS was established in 1975 as a fatal motor vehicle accident reporting system for all 50 states, the District of Columbia, and Puerto Rico. Crashes must have resulted in the death of a motorist or a non-motorist within 30 days of the collision to be entered into FARS. Under strict quality control procedures, trained NHTSA analysts extract data from state report files including police crash reports, death certificates, state vehicle registrations, coroner/medical examiner reports, state driver licensing files, state highway department data, emergency medical service reports and vital statistics, and other state records to collect information relating to the crash, vehicles, and people involved [27]. Although FARS has been recording alcohol tests since 1975 and non-alcohol drug tests since 1991, there is not a consistent standard or method for conducting and reporting drug tests results across the states. For alcohol tests, NHTSA used multiple imputations to allow further analyses on blood alcohol concentration (BAC) [28].

### Study sample

The study sample was restricted to states that conducted toxicological testing for non-alcohol drugs for more than 50% of all fatally-injured drivers each year between 2007 and 2017. This restriction ensured that inconsistency of drug test data across states and years was limited. In addition, we restricted fatally-injured drivers to those who were at least 16 years old and died within 1 h of the vehicle crash because testing is more common in such instances and therefore results in more reliable data. Based on our inclusion criteria, our analytic sample included 102,221 drivers from the following 33 states: Arizona, California, Colorado, Connecticut, Florida, Georgia, Hawaii, Illinois, Indiana, Kentucky, Louisiana, Maryland, Massachusetts, Michigan, Minnesota, Missouri, Montana, Nevada, New Hampshire, New Jersey, New Mexico, New York, North Dakota, Ohio, Pennsylvania, Rhode Island, South Carolina, Tennessee, Vermont, Virginia, Washington, West Virginia, and Wisconsin. Overall, these states performed toxicological testing on more than 80% of their fatally-injured drivers for the combined years (2007 to 2017).

### Measures

Toxicological drug tests results were based on the blood or urine of fatally-injured drivers. For each driver, up to three drug test results were recorded for non-alcohol drugs. FARS documents drugs in the following categories: narcotic, depressant, stimulant, hallucinogen, cannabinoid, phencyclidine, an anabolic steroid, inhalant, and other drugs. We defined a tested-positive driver as a driver with at least one positive test result for one of the listed categories. A tested-negative driver was considered a driver whose test results were negative for all drug tests taken. Among those who tested positive for drugs, we examined the status of alcohol tests and derived three categories: drug positive and alcohol-negative, drug positive and alcohol positive, and drug positive and alcohol unknown. For drivers aged between 16 and 20 years, if test results were at or above 0.02 g per deciliter, alcohol test results were considered positive given that the majority of states in our study use this BAC level as per se evidence for impaired driving. For drivers over 21 years of age, we used the standard 0.08 g per deciliter as the threshold for a positive alcohol test.

We also obtained the drivers’ characteristics including age, sex, race, Hispanic origin, state, information about whether death occurred at the scene or on the way to a hospital, blood alcohol concentration from an alcohol test, year of death, month of death, hours between time of the crash and time of death, and drug test results. Age was categorized as 16–20, 21–44, 45–64, and 65+. Race and Hispanic origin were recoded as non-Hispanic White, non-Hispanic Black, Hispanic, and other race groups. We used death at the scene and death during transport in our analysis, as such information is readily available in FARS. BAC was categorized as 0, 0.001–0.079, and ≥ 0.08.

### Statistical analysis

Demographic characteristics were described, using aggregated prevalence from 2007 to 2017 (Table 1), by the drug test results status (tested positive or tested negative) of drivers who died within 1 h of the vehicle crash. Categorical variables were reported with frequency and relative frequency (%). Chi-Square tests were performed to assess significant differences in demographic characteristics among groups and corresponding test-statistic values, degrees of freedom (d.f.), and *p*-values were provided. We generated estimates of the yearly prevalence of drivers for drug test results for each year of data for the full sample as well as estimates by sex, race, and age subgroups (see Additional file 1). Additionally, we plotted estimates of the yearly prevalence of drug test-positive drivers for the full sample as well as estimates by sex, race, and age subgroups. In the trend analyses, Cochran–Armitage tests were used to assess the statistical significance of changes in the yearly prevalence of drug involvement in fatally-injured drivers over time. We also conducted Cochran–Armitage tests for each subgroup. All tests were two-sided, and a *p*-value of < 0.01 was considered significant given the large sample size of our data. The Z test-statistics values corresponding to the Armitage tests were also provided.

**Table 1:** Characteristics of fatally-injured drivers who died within 1 h of crashing, by drug test results, aggregated prevalence from 2007 to 2017

Demographic Characteristics^e^	Tested Positive	Tested Negative	Other^d^	Total	Chi-square test
	n (%^a^)	n (%^a^)	n (%^a^)	n (%^b^)	χ^2^, d.f., *p*-value
	28,756 (28.1)	49,384 (48.3)	24,081 (23.6)	102,221 (100.0)	
Age					1126.3,6,<.0001
16–20	2433 (25.7)	4876 (51.4)	2176 (22.9)	9485 (9.3)	
21–44	15,893 (31.6)	23,275 (46.3)	11,107 (22.1)	50,275 (49.2)	
45–64	8241 (27.7)	14,317 (48.2)	7150 (24.1)	29,708 (29.1)	
65+	2189 (17.2)	6916 (54.2)	3648 (28.6)	12,753 (12.5)	
Sex					21.6,2,<.0001
Male	22,609 (28.4)	38,470 (48.3)	18,526 (23.3)	79,627 (77.9)	
Female	6142 (27.2)	10,904 (48.3)	5548 (24.6)	22,594 (22.1)	
Race					405.9,6,<.0001
Non-Hispanic White	20,572 (30.2)	31,891 (46.8)	15,747 (23.1)	68,210 (72.3)	
Non-Hispanic Black	3011 (26.6)	5535 (48.8)	2795 (24.7)	11,341 (12.0)	
Hispanic	2760 (24.3)	6292 (55.5)	2292 (20.2)	11,344 (12.0)	
Other Race	855 (24.8)	1884 (54.7)	706 (20.5)	3445 (3.7)	
Died at Scene/En Route					23.8,6, <.01
Not Applicable^c^	5039 (28.0)	8843 (49.1)	4134 (23.0)	18,016 (17.6)	
Died at Scene	23,420 (28.1)	40,105 (48.2)	19,774 (23.7)	83,299 (81.5)	
Died En Route	289 (33.2)	415 (47.7)	166 (19.1)	870 (0.9)	
Blood Alcohol Concentration					867.2,6,<.0001
0	15,601 (30.5)	30,879 (60.4)	4326 (9.1)	51,106 (58.8)	
0.001–0.079	1978 (41.6)	2279 (47.9)	502 (10.6)	4759 (5.5)	
≥ 0.08	10,742 (34.6)	15,872 (51.1)	4430 (14.3)	31,044 (35.7)	
Month of Death					163.2,22,<.0001
1	2069 (28.1)	3614 (49.1)	1675 (22.8)	7358 (7.2)	
2	1896 (28.5)	3299 (49.6)	1461 (22.0)	6656 (6.5)	
3	2241 (28.1)	3972 (49.7)	1773 (22.2)	7986 (7.8)	
4	2440 (28.8)	4157 (49.1)	1876 (22.1)	8473 (8.3)	
5	2641 (28.7)	4518 (49.1)	2041 (22.2)	9200 (9.0)	
6	2609 (28.8)	4362 (48.1)	2103 (23.2)	9074 (8.9)	
7	2712 (28.2)	4697 (48.8)	2209 (23.0)	9618 (9.4)	
8	2694 (27.7)	4767 (49.0)	2266 (23.3)	9727 (9.5)	
9	2562 (28.5)	4264 (47.5)	2149 (23.9)	8975 (8.8)	
10	2476 (27.5)	4334 (48.2)	2179 (24.2)	8989 (8.8)	
11	2281 (27.8)	3796 (46.2)	2134 (26.0)	8211 (8.0)	
12	2135 (26.8)	3604 (45.3)	2215 (27.8)	7954 (7.8)	

^a^ % = row percentage

^b^ % = column percentage

^c^ Not Applicable is used for victims dying at locations other than the scene or en route (e.g. Hospital, at home, etc.)

^d^ The other group included fatally-injured drivers whose test results were unknown or not reported and drivers who were not given drug tests

^e^ Chi-Square tests were performed to assess significant differences in demographic characteristics among groups and all differences were significant (*p* < 0.01)

There were so many individuals in the “unknown” category of intoxication for each of the demographic variables and therefore we examined if those individuals were randomly distributed between the intoxicated and non-intoxicated group. This examination was done by generating a descriptive table for demographic characteristics by the drug test results status (Known vs. unknown) and then calculating the effect size (Cohen’s h) for the difference in characteristics between the two groups. All effect sizes were < 0.25, which indicates a very small difference (see Additional file 1) considering that h = 0.20: “small effect size”; h = 0.50: “medium effect size”; and h = 0.80: “large effect size”. In addition, we treated the unknown test results as missing and used chi-square test of the missing completely at random (MCAR) mechanism to determine the missing data were completely at random (*P* > 0.99) [29]. We performed all data analyses using SAS version 9.4 (SAS Institute, Inc., Cary, NC).

## Results

Characteristics of drivers who died within 1 h of crashing are reported according to drug test result in Table 1 using aggregated prevalence from 2007 to 2017. Of all 102,221 drivers included in the analysis, 28,756 (28.1%) tested positive for non-alcohol drugs and 49,384 (48.3%) tested negative for non-alcohol drugs. Out of all drivers, 77.9% were male, 81.5% died at the scene, 72.3% were non-Hispanic White, and 58.5% were younger than 45 years old. The number of drivers for each year ranged from 7901 to 10,839. Compared to female fatally-injured drivers, male fatally-injured drivers were more likely to have a positive drug test result (28.4% vs 27.2%) but less likely to have other drug test results (23.3% vs 24.6%).

**Fig. 1:**
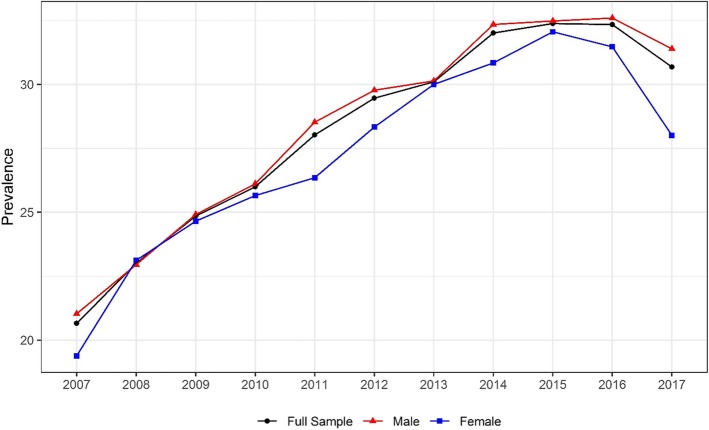
Trend in yearly prevalence in positive drug test among drivers who died within 1 h of crashing, by sex. Cochran–Armitage tests were used to assess the statistical significance of changes in the percentage of drug involvement in fatally-injured drivers over time (Male:Z = 23.7,*p* < 0.001;Female: Z = 11.4,*p* < 0.001)

The yearly prevalence of the three-drug test types is shown in Table 2 for those drivers who died within 1 h of crashing for each year from 2007through 2017. The yearly prevalence of drug-positive drivers increased significantly from 20.7% in 2007 to 30.7% in 2017. Figure 1 presents the yearly prevalence of drug-positive drivers by sex. For all years except 2008, yearly prevalence of drug-positive drivers among female fatally-injured drivers was higher than males. In both sexes, yearly prevalence of drug-positive drivers increased significantly from 21.0 and 19.4%, respectively, in 2007 to 31.4 and 28.0% in 2017. However, between 2015 and 2017, the yearly prevalence of female tested-positive drivers decreased from 32.0 to 28.0%. In 2009, the difference between yearly prevalence in males and females began to widen, with males displaying noticeably higher rates of death within 1 h of a crash (Fig. 1). This continued until 2013, when the gap closed, and women had a similar yearly prevalence to men. The difference again expanded after 2013.

**Table 2:** Yearly prevalence of drug test result among fatally-injured drivers who died within 1 h of crashing by year, 2007 to 2017

Death Year	Full Sample	Tested Positive		Tested Negative		Other*	
	n	n (%)		n (%)		n (%)	
2007	10,839	2239	20.70%	5786	53.40%	2814	26.00%
2008	9642	2218	23.00%	5186	53.80%	2238	23.20%
2009	8362	2078	24.90%	4297	51.40%	1987	23.80%
2010	8029	2087	26.00%	4359	54.30%	1583	19.70%
2011	7901	2214	28.00%	4094	51.80%	1593	20.20%
2012	8969	2642	29.50%	4345	48.40%	1982	22.10%
2013	8868	2669	30.10%	4146	46.80%	2053	23.20%
2014	8720	2791	32.00%	4029	46.20%	1900	21.80%
2015	9838	3185	32.40%	4477	45.50%	2176	22.10%
2016	10,533	3406	32.30%	4756	45.20%	2371	22.50%
2017	10,520	3227	30.70%	3909	37.20%	3384	32.20%
Cochran–Armitage test Z, P-value**		Z = 26.3, *p* < .0001		Z = -29.3, p < .0001		Z = 6.7, p < .0001	

*The other group included fatally-injured drivers whose test results were unknown or not reported and drivers who were not given drug tests. **Cochran–Armitage tests were used to assess the statistical significance of changes in the percentage of drug involvement in fatally-injured drivers over time (p < 0.01)

**Fig. 2:**
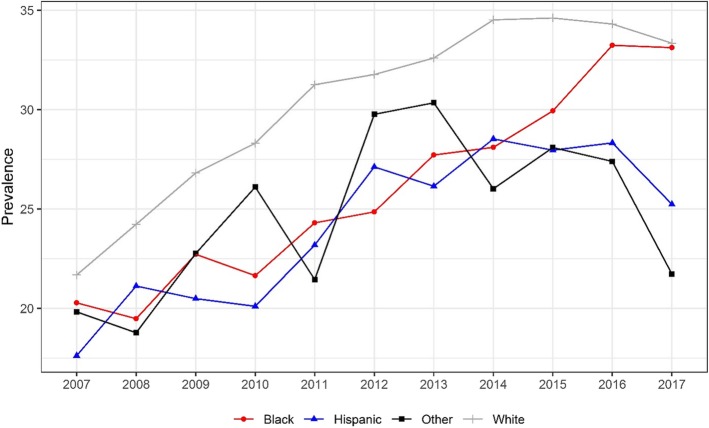
Trend in yearly prevalence in positive drug test among drivers who died within 1 h of crashing, by race. Cochran–Armitage tests were used to assess the statistical significance of changes in the percentage of drug involvement in fatally-injured drivers over time (White: Z = 23.5,*p* < 0.001;Black: Z = 11.6,*p* < 0.001;Hispanic: Z = 8.0,*p* < 0.001;Other: Z = 2.7,*p* < 0.01)

Figure 2 shows the 2007–2017 trends in drug-positive driver yearly prevalence by race. The yearly prevalence of drug-positive drivers was highest in non-Hispanic Whites than other race categories. In all race subgroups, the yearly prevalence increased significantly from 2007 to 2017. In the non-Hispanic White and Hispanic groups, between 2014 and 2017, the yearly prevalence decreased from 34.5 and 28.5% to 33.3 and 25.2%, respectively. The gap between non-Hispanic White and Black drug-positive fatality percentage was considerable but narrowed significantly over time. The difference between the two rates peaked in 2011 when the non-Hispanic White yearly prevalence was approximately 6.9% higher than the Black yearly prevalence. In 2017, this difference had narrowed, and White yearly prevalence was 0.2% higher than the Black yearly prevalence.

**Fig. 3:**
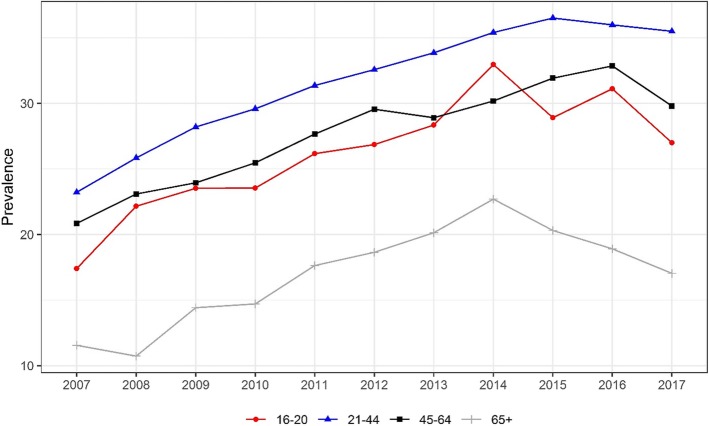
Trend in yearly prevalence in positive drug test among drivers who died within 1 h of crashing, by age. Cochran–Armitage tests were used to assess the statistical significance of changes in the percentage of drug involvement in fatally-injured drivers over time (16–20: Z = 8.5, *p* < 0.00; 21–44: Z = 20.5, *p* < 0.001; 45–64:Z = 13.4, *p* < 0.001; 65+: Z = 8.0, *p* < 0.001)

The yearly prevalence of drug-positive tests by age group are reported in Fig. 3. Drug-positive tests were more prevalent in the 21–44 age group and lowest in the 65+ age group. The yearly prevalence increased significantly in all age groups from 2007 to 2017. However, the percentage of those aged 16–20 decreased from 28.9% in 2015 to 27.0% in 2017. For those aged 65+, the yearly prevalence peaked at 22.7% in 2014 and subsequently declined. There was a decline in yearly prevalence of all other age groups from 2016 to 2017.

**Fig. 4:**
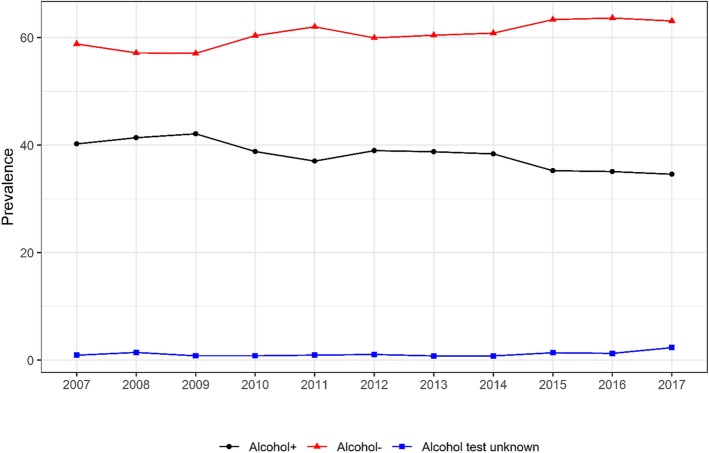
Trend in yearly prevalence in alcohol test result among drug-positive drivers who died within 1 h of crashing. Cochran–Armitage tests were used to assess the statistical significance of changes in the percentage of alcohol involvement in drug tested positive fatally-injured drivers over time (Alcohol+: Z = 7.5, *p* < 0.001; Alcohol-: Z = -6.6, *p* < 0.001; Alcohol test unknown: Z = -3.9, *p* < 0.001)

Among drug-positive drivers, Fig. 4 shows the alcohol test result status from 2007 to 2017. The yearly prevalence of alcohol negative tests was higher than alcohol positive tests. Yearly prevalence of alcohol test negative and alcohol test unknown subcategories increased from 2007 to 2017, while the yearly prevalence of alcohol-positive test decreased. Among drivers who tested positive for drugs in 2017, 63% had a BAC below the threshold of per se evidence for impaired driving, and 34.6% had a BAC above the threshold.

## Discussion

While driving under the influence of alcohol is a well-recognized public health hazard, less attention is paid to other drugs. The last two decades in the U.S. have witnessed a tremendous shift in the norms and perception regarding substance use (e.g. marijuana) in addition to the ongoing opioid epidemic. Such cultural changes represent a variety of potential health hazards, including drugged driving. The present study augments the literature by examining the yearly prevalence of drug-positive drivers from 2007 to 2017 in the general population, as well as analyzing trends among subgroups. Trend analyses were performed to detect changes in the yearly prevalence of drug-positive drivers over time.

The yearly prevalence of fatally-injured drivers who tested positive for drugs increased significantly from 2007 to 2017, with the highest rate of 32.4% occurring in 2015. This coincides with the U.S. opioid epidemic, which, since 1999, has seen continual increases in abuse of prescription opioids, heroin, and synthetic opioids [30]. Our findings are in keeping with what has been reported elsewhere, including hospital emergency data and studies from different countries. There is evidence of high rates of drug use among drivers hospitalized following motor vehicle crashes. For example, one study reported that 61.5% of drivers admitted to a trauma center after an accident reported use of prescription medications [31] and another found that 51% of drivers admitted to a trauma center for vehicle crash injuries tested positive for drugs other than alcohol, with 25% testing positive for marijuana use [32]. A study conducted in Canada found that marijuana and other drugs are now commonly detected in injured drivers [33]. Similar findings have been reported in other countries, including Australia [34, 35], Belgium [36], and Spain [37].

Our results showed that the yearly prevalence of positive drug tests in fatally-injured drivers was consistently higher among males than females from 2007 to 2017, the exception being 2013. These results, in part, could be explained by gender risk perception differences, with men less likely to perceive dangerous driving habits as serious [38]. Prior research points to a positive association between masculinity and the number of offences and aggressive violations [39]. In fatal road crashes involving loss of control due to speed, males were five times more likely to be at fault than their female counterparts [40]. Males have also displayed higher fatality rates than females for most modes of travel [41]. In a study examining traffic-related head trauma emergency department visits, the majority of patients were male [42].

We found that out of all fatally-injured drivers included in the analysis, a vast majority were non-Hispanic White. In the U.S., White residents have higher rates of opioid overdose death compared with other races. Similarly, heroin use by race has shifted in the last 50 years from a primarily inner-city, minority-centered issue to a more widespread problem involving mostly White men and women living in non-urban areas [43]. Our results agree with these previous studies, i.e., the percentage of positive drug tests was substantially higher among White drivers compared with the other racial groups. However, the gap between White and Black percentage narrowed significantly over time.

Previous findings have shown that when compared to other age groups, 16–17 year olds are predisposed to and consistently display the highest rates of involvement in all police-reported crashes, and 80+ year olds have the highest rates of fatal crash involvements [44]. We found that the yearly prevalence of fatally-injured drug-positive drivers was consistently highest in the 21–44 age group compared to other age groups. Possible contributors are the facts that in the U.S., 21–25 year olds display some of the highest rates of illicit drug use [45] and drivers aged 20–49 make up a large portion of all drivers [46]. National statistics show that from 2010 to 2011, 16–24 year olds had the highest rates of emergency department visits for motor vehicle traffic injuries, followed by those aged 25–44 years old [47]. Although there was a decline in yearly prevalence in all age groups between 2016 and 2017, the 21–44 age group showed the smallest change.

Evidence suggests that while alcohol-impaired driving has witnessed a decreasing trend, at least in some jurisdictions, drugged driving appears to be increasing [48]. Among drug-positive fatally-injured drivers, the majority tested negative for alcohol (below the BAC threshold for per se evidence for impaired driving). This further highlights that drugged driving remains a growing public health concern, and more action is needed to curb this trend. While fewer drug-positive drivers had alcohol above the acceptable BAC level for driving, it should be noted that concurrent use of drugs can lead to more deleterious effects [49]. The BAC needed for a fatal overdose is lower when alcohol and prescription drugs are used simultaneously than when alcohol is used alone [50]. One study identified an increase from 2005 to 2011 in the number of U.S. emergency department visits due to adverse drug reactions that also involved alcohol [51].

Our results and previous findings demonstrate that drugged driving is a public health issue. With more states legalizing marijuana and the ongoing opioid epidemic, the issue is likely to amplify in the coming years. Currently, in the U.S., states have started implementing drugged driving laws, and these laws vary accordingly in many aspects. While promising, it remains unclear what impact these laws will have on drugged driving and related adverse outcomes such as traffic crashes and fatalities. Future research may consider expanding our findings by investigating differences in motor vehicle accident rates and related outcomes between states, as the legality of marijuana varies by state. In addition, while there is an established body of evidence surrounding the scientific evidence for alcohol per se laws, research is lacking for implementation of a scientifically supported threshold for drugged driving. It is important to note that this a potentially a complex issue given individual variability in the levels of tolerance or intoxication [52, 53].

This study had a few limitations. There is no standardized testing process for drugged driving; therefore, reporting across states and jurisdictions is not uniform. However, we limited potential bias due to a lack of standard reporting guidelines across the states by analyzing data only from those states that conducted toxicological testing for non-alcohol drugs for more than 50% of all fatally-injured drivers each year. Though FARS applies strict quality control procedures, there is a possibility of reporting errors. Another possible limitation is that the cause of death is unknown; whether the individual died from the drug or the accident is unclear, and the current study makes no claim about the cause of death. Despite the given limitations, the present study investigated important differences in the aggregated prevalence and characteristics of fatally-injured drug-positive drivers over an entire decade, from 2007 to 2017.

## Conclusion

Using data from FARS, this study investigated the yearly prevalence of drug-positive drivers each year from 2007 through 2017 in the general population as well as by key demographic characteristics (sex, race, and age). Of fatally-injured drivers who died within 1 h of a vehicle crash and were included in our analysis, a large majority were non-Hispanic White, male, died at the scene of the accident, and had a BAC level of less than 0.001. Overall, the yearly prevalence of fatally-injured drivers who tested positive for drugs increased significantly from 2007 to 2017. Positive drug tests were more prevalent in males, those age 21 to 44, and non-Hispanic Whites. The majority of drug-positive drivers had a BAC under the threshold for per se evidence of alcohol-impaired driving. Our findings further highlight that drugged driving continues to grow as a public health concern and action should be taken to curb this troubling trend.

## Supplementary information

**Additional file 1: Table S1.** Characteristics of fatally-injured drivers who died within 1 hour of crashing, by drug test results, 2007 to 2017. **Table S2.** percentage of drug test result among fatally injured drivers who died within 1 hour of crashing by year and sex, 2007 to 2017. **Table S3.** percentage of drug test result among fatally injured drivers who died within 1 hour of crashing by year and race, 2007 to 2017.**Table S4.** percentage of drug test result among fatally injured drivers who died within 1 hour of crashing by year and age, 2007 to 2017.

## Data Availability

The data is publicly available.

## Abbreviations

BAC: Blood alcohol Concentration
FARS: Fatality Analysis Reporting System
NHTSA: National Highway Traffic Safety Administration
